# The effect of neuroticism on depressive symptoms in Chinese college students: maternal parenting practices as moderators

**DOI:** 10.3389/fpsyg.2025.1584212

**Published:** 2025-06-20

**Authors:** Bao Zhao, Xiaoyu Wang, Xinyao Jiang, Ruixue Zhuang, Jiaqi Li, Nian Ji, Dengting Boyanton

**Affiliations:** ^1^School of Psychology, Shandong Normal University, Jinan, Shandong, China; ^2^Collaborative Innovation Center of Assessment toward Basic Education Quality, Beijing Normal University, Beijing, China; ^3^Columbus Academy, Gahanna, OH, United States; ^4^Student Mental Health and Education Center, Shandong Normal University, Jinan, Shandong, China; ^5^171 SIG Chair, American Educational Research Association (AERA), Washington, DC, United States

**Keywords:** neuroticism, depressive symptoms, college students, maternal parenting practices, moderation

## Abstract

**Background:**

Depressive symptoms is extremely prevalent in college students nowadays. It can cause long-term suffering and may even lead to suicidal ideation. It has been indicated by research that depression is related to a variety of psychosocial factors, the most notable being neuroticism and parenting. However, the underlying mechanisms of these variables have remained unclear. The purpose of the current study was to investigate the interaction between maternal parenting practices and neuroticism and its effects on depression.

**Method:**

A total of 2,692 undergraduate students were enrolled in this cross-sectional investigation from four universities located in Shandong Province, China. Participants filled simplified versions of the Big Five Personality Inventory, Parental Bonding Instrument, and Self-Rating Depressive Symptoms Scale. After eliminating entries with incomplete values, the dataset comprised 2,588 complete responses for analysis. Structural equation modeling (SEM) was performed using SPSS 22.0 and the PROCESS macro for data analysis.

**Results:**

The results indicated that both neuroticism (*r* = 0.572, *p* < 0.001) and maternal control factor (*r* = 0.253, *p* < 0.001) displayed strong positive correlation with depression; whereas maternal care factor (*r* = −0.402, *p* < 0.001) and maternal encouraging autonomy factor (*r* = −0.345, *p* < 0.001) are negatively correlated with depression. Additionally, neuroticism demonstrated a significant direct effect on depression (β = 0.571, *p* < 0.001). Moderation models were employed to examine the relationship between depression, maternal parenting practices, and neuroticism. Specifically, a high level of maternal care (Δ*R*^2^ = 0.001, *p* = 0.046) and maternal encouraging autonomy (Δ*R*^2^ = 0.0046, *p* = 0.004) significantly weakened the connections between neuroticism and depression, while an elevated level of maternal control enhanced the relationship between neuroticism and depression (Δ*R*^2^ = 0.0019, *p* = 0.038).

**Conclusion:**

This study presents initial evidence for the moderating role of maternal parenting practices in the neuroticism-depression association. These results may facilitate the development of targeted intervention protocols tailored to university student subgroups based on different socioeconomic demographic characteristics and personality profiles.

## 1 Introduction

Depressive symptoms are widespread among the contemporary college students, encompassing affective states such as persistent sadness, hopelessness, anxiety, loss of interest, and even suicidal thoughts (Cui et al., 2024; Choi et al., 2019). College students represent a vulnerable population due to challenges associated with identity formation, role transitions, and adaptations to evolving lifestyle and learning demands (Liu et al., 2019). Studies demonstrate significantly higher prevalence of depressive symptoms than the general populations (Liu et al., 2023; Zhang et al., 2022), with evidence suggesting an increase in incidence (Zhang et al., 2022). Notably, Chinese college students face exacerbated academic and employment pressures under the influence of Asian cultural values, with recent studies documenting an alarming rise in depressive prevalence within this cohort (Liu et al., 2023). Depression can significantly impair academic performance, interpersonal functioning, and long-term career trajectories (Choi et al., 2019; Wang et al., 2020; Gao et al., 2020). These severe consequences and unique contextual stressors underscore the critical need to investigate mechanisms and risk factors underlying depression among Chinese university students. Consequently, this study examines contributing factors to depression in Chinese university students, a population demonstrating elevated depression risk relative to the general public (Zhang et al., 2022).

Neuroticism, a personality trait marked by increased susceptibility to negative emotional reactions when facing setbacks, failures, and perceived threats (Lahey, 2009), has been consistently found to be a key risk factor for depression across numerous studies (Kendler et al., 2004; Kotov et al., 2010; McDonnell and Semkovska, 2020; Muris et al., 2005; Sheldon et al., 2021; Wilks et al., 2020; Yang and Koo, 2022; Yuan, 2011). This positive correlation between neuroticism and depression has also been empirically validated in research involving Chinese college students (Yang et al., 2023; Yu et al., 2023), making it necessary to re-examine the relationship within this particular group and look further into the underlying mechanisms.

Research indicates that parenting practices likely play a key role in modulating the onset and trajectory of depressive symptoms(McLeod et al., 2007; Kaslow et al., 1994). In China, maternal figures predominantly assume primary childcare responsibilities, whereas paternal involvement in child-rearing remains comparatively limited (Xu and Wang, 2019). This caregiving asymmetry results in mothers engaging in more frequent interactions with their offspring and exerting greater psychosocial influence compared to fathers or alternative caregivers (Liu et al., 2021). Empirical evidence underscores the particularly salient impact of maternal parenting practices on mental health outcomes across developmental stages, from childhood through adolescence (Chubar et al., 2020). Longitudinal studies further reveal dynamic associations between depressive symptoms and maternal parenting patterns during middle-to-late childhood (Wu et al., 2023). Nevertheless, scant research has specifically investigated the effects of maternal parenting on mental health within the Chinese college student population. This critical gap motivates our systematic examination of maternal parenting practices contributions to depression among undergraduate students.

The diathesis-stress model offers a comprehensive framework for understanding depression, emphasizing the interplay between internal vulnerabilities (diathesis) and external stressors (Monroe and Simons, 1991). Originally formulated in the 1960s to explain schizophrenia etiology, this model was later adapted to depression research during the 1980s. This model posits that stress activates an underlying diathesis, transforming a latent predisposition into psychopathology (Xu, 2018). The interaction between diathesis and stress is synergistic, producing effects greater than their individual contributions, with both additive and multiplicative influences on depression risk (Monroe and Simons, 1991). Internal diathesis refers to stable, developmental traits such as personality or temperament (Cloninger et al., 1994). These factors increase susceptibility to stress and depression in three key ways: (1) diathesis alone may suffice, with stress merely exacerbating preexisting vulnerability; (2) severe stress alone can trigger depression in the absence of strong diathesis or (3) diathesis and stress jointly constitute necessary conditions for depression (Belsky, 1997). As the primary interface through which individuals interact with their environment (Ebrahimi et al., 2017), families exert profound developmental impacts. According to this model, maternal parenting may moderate the neuroticism-depression relationship as an important familial environmental factor (Monroe and Simons, 1991), though the precise mechanisms underlying this moderation remain understudied.

Within this framework, family influences operate bidirectionally. A retrospective view of existing research shows that most studies emphasize the vulnerability caused by personality factors. For instance, self-criticism interacting with adverse life events (Mendelson and Gruen, 2005) or perceived stress (Robillard et al., 2021) leads to depression. Studies based on the diathesis-stress model have confirmed that family risk and early life stress are linked to depression in children and adolescents (Nielsen et al., 2020). A study on Chinese children indicates that children with “risky" temperamental traits are more likely to experience maladjustment under unfavorable parenting conditions (Xia et al., 2017). However, little research has targeted the population of neurotic Chinese college students. Conversely, positive parenting practices—particularly those fostering self-confidence and constructive self-perception (Katz and Hunter, 2007; Gaté et al., 2013)—may serve as potent protective factors. Empirical studies have identified harmonious family relationships as protective factors against risky behaviors in neurotic adolescents (Yuan, 2011), and positive parenting practices as buffers for youth with neurological conditions during the COVID-19 pandemic (Green et al., 2024). Recent research on Chinese university students consistently emphasizes the buffering effect of perceived social support on depressive symptoms (Yang and Lei, 2024; Yu and Hu, 2022). However, no studies have explicitly conceptualized familial influences as parenting practices, and the differentiated moderating effects on the neuroticism-depression relationship in Chinese college students have not been examined.

Additionally, Multiple studies demonstrate that family socioeconomic status (SES) negatively correlates with depression among Chinese college students (Gang and Dajun, 2018; Xie, 2020; Li, 2024). The role of SES is predominantly explored via three key variables: educational qualification, occupational status, and income levels (Klimesch et al., 2024). Specifically, parental education not only reduces the risk of depression in students (Zhao et al., 2015) but also enhances psychological wellbeing and self-esteem (Zhao, 2017). In contrast, family income shows inconsistent effects—some studies find no significant impact (Zhao et al., 2015; Xie, 2020), whereas others highlight its critical role (Xie, 2020). Thus, this study intends to explore the relationship between each sub-dimension of SES and the depressive symptoms of college students.

**Figure 1:**
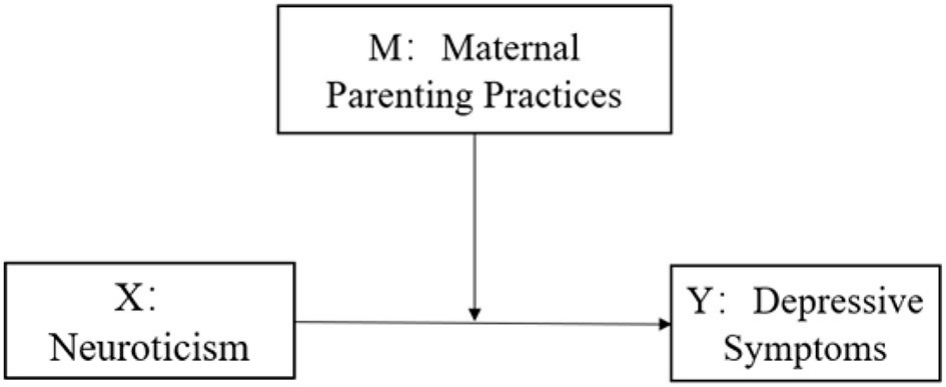
The hypothesized model (The moderation effect analyses were conducted across three dimensions of maternal parenting practices).

Summarizing, significant gaps persist in understanding the moderating role of maternal parenting practices in the neuroticism-depression relationship. While extensive research has explored the association of neuroticism and depression, maternal parenting practices remain under-examined as moderators, particularly among Chinese college students. No prior studies have empirically demonstrated interactions between neuroticism and maternal parenting practices in predicting depression within this population, nor clarified the mechanisms linking depression to the three dimensions of maternal parenting practices (maternal care, maternal encouraging autonomy, and maternal control), necessitating rigorous investigation into these dynamics. To address these gaps, this study proposes a conceptual moderation model (Figure 1) with three hypotheses: 1) Neuroticism significantly predicts depression in Chinese college students; 2) Maternal care and maternal encouraging autonomy negatively correlate with depression, whereas maternal control exhibits positive predictive effects; 3) Maternal parenting practices moderate the correlation between neuroticism and depressive symptoms in college students.

## 2 Methods

### 2.1 Participants

This investigation utilized a convenience sampling method to recruit 2,692 college students from four universities in Shandong Province, China. Data was collected between May and June 2017. The required sample size was determined via G*Power software (v3.1) using a linear multiple regression model (fixed model, *R*^2^ increase). Parameters included a small effect size *f*^2^ = 0.02 for the interactive term (one tested predictor, total number of predictors = 3, α = 0.05, powe*r* = 0.80). A sample size of 395 would be necessary to adequately detect the moderation effect (Faul et al., 2007). Questionnaires with substantial missing values were systematically excluded during initial screening. Low-quality data were defined as cases with either invariant responses (e.g., identical choices on consecutive items) or logical inconsistencies, all of which were eliminated after systematic screening of response patterns. 2,588 valid questionnaires were collected after screening (the recovery rate is 96.14%). Participants consisted of 2,588 Chinese students (80.1% female), enrolled in different majors. Participants had a mean age of 19.6 years (range: 16-26). The average age of the participants' mothers was 46.3 years (ranging from 35 to 67 years), and the mean age of the participants' fathers was 46.9 years (ranging from 35 to 67 years).

### 2.2 Procedures

Ethical approval for this research was obtained from the Ethics Committee of the university that the authors are affiliated with. Prior to data collection, trained research personnel provided participants with detailed instructions regarding voluntary participation, confidentiality protocols, and the right to withdraw without consequence. After the acquisition of informed consent, participants were invited to fill out the questionnaires under supervised conditions to ensure independence and confidentiality of the responses. The participants accomplished the simplified version of the Big Five Personality Inventory, the Self-Rating Depression Scale, and the Parental Bonding Instrument (PBI; Chinese version). The participants were warranted of the confidentiality of the information they provided, and the autonomy of their responses was affirmed by the research assistant. Ethical approval was attained from Shandong Normal University (Permit Number: SDNU2019069).

### 2.3 Measurements

#### 2.3.1 Neuroticism

The simplified Big Five Personality Inventory (NEO-FFI: Neuroticism, Extraversion, Openness Five-Factor Inventory), developed by Costa and McCrae (2008), assesses five personality traits (conscientiousness, neuroticism, extraversion, openness, agreeableness). Each dimension is measured via 12 items on a 5-point Likert scale (1 = strongly disagree; 5 = strongly agree). Validation studies confirmed its applicability for Chinese university populations (Yao and Liang, 2010). The neuroticism subscale demonstrated an internal consistency reliability of 0.85, with the full scale at 0.72.

#### 2.3.2 Parenting practices

Maternal parenting practices were evaluated using the Chinese adaptation of the Parental Bonding Instrument (PBI; Parker, 1981), validated for Chinese college students by Yang et al. (2009). The PBI is a self-report scale designed to evaluate retrospective perceptions of parental behaviors before age 16. Respondents rated statements on a 4-point scale (0 = strongly disagree; 3 = strongly agree). The instrument includes both the maternal version (PBI-M) and the paternal version (PBI-F); each comprises 23 items categorized into three dimensions: care, encouraging autonomy, and control. The PBI-M was applied in the present study with a Cronbach's α of 0.68.

#### 2.3.3 Depression

Depressive symptoms were quantified via the Zung Self-Rating Depression Scale (SDS; Zung, 1965), comprising 20 items, validated for Chinese college students by Liu et al. (1995). Respondents selected frequency levels (never/occasionally, sometimes, often, always) on a 4-point scale. The severity score of depression was computed by aggregating the scores of all items, with higher values indicating more significant depressive tendencies. The SDS standard score has a cutoff of 53 points, where scores of 53–62 suggest mild depression, 63–72 indicate moderate depression, and over 72 signify severe depression (Wang and Chi, 1984; Wang et al., 1986). The Cronbach's α in the current study was 0.85.

#### 2.3.4 Family socioeconomic status

Family socioeconomic status (SES) is composed of parents' educational levels, parents' occupations, and family income (Zhang et al., 2021). Parents' occupations are categorized into three classes based on their professional and technical nature: “farmers or unemployed individuals" (scored as 1), “semi-professionals" (scored as 2), and “professionals" (scored as 3). Parents' educational levels are divided into six categories and then grouped into three classes: those with education below high school are scored as 1, high school education is scored as 2, and education above college level is scored as 3. Family monthly income is categorized into three groups: below 3,000 Chinese Yuan is scored as 1; 3,000 to 6,000 Chinese Yuan is scored as 2; and above 6,000 Chinese Yuan is scored as 3.

### 2.4 Data analyses

All statistical procedures—including descriptive statistics, correlation analyses, common method bias assessment, and model testing for moderation effect—were conducted suing SPSS 22.0 and PROCESS macro (Hayes, 2013). The correlations among variables were analyzed with SPSS 22.0. Subsequently, three simple moderation analyses were carried out with neuroticism as the independent variable and college students' depression as the dependent variable. The three dimensions of maternal parenting practices, namely maternal care, maternal encouraging autonomy, and maternal control, were entered as moderators. Finally, a moderation analysis (simple slope analysis) was conducted to explore how maternal parenting practices moderated the association between neuroticism and college students' depression. The bootstrap method was employed to test the confidence intervals (CI) for the proposed model, with 1,000 resampling iterations and Model 1 selected for analysis (Hayes, 2013). Due to the significant correlation between SES and other variables, SES was controlled in all moderation analyses. Additionally, age and gender were controlled for in all correlation and regression analyses. To reduce the false discovery rate, we applied the correction procedure based on a simple sequential Bonferroni type (Benjamini and Hochberg, 1995).

## 3 Results

### 3.1 Sample characteristics

The profile of the participants was presented in the Table 1.

**Table 1:** Sample characteristic.

**Variable**	**Options**	**Number**	**Percentage (%)**
Age	16–26 years	2,588	100.0
Gender	Male	514	19.9
	Female	2,074	80.1
Grade	Freshmen	1,578	61.0
	Sophomores	733	28.3
	Juniors	246	9.5
	Seniors	31	1.2
Major	Science	1,053	40.7
	Liberal arts	1,104	42.7
	Arts	148	5.7
	Engineering	283	10.9

The details regarding the parents of the participants were provided in Table 2.

**Table 2:** SES information.

**SES**	**Options**	**Parents**	**Percentage (%)**
Education background of parents	High School and Below	Fathers	54.4
		Mothers	65.8
	A high school education	Fathers	25.7
		Mothers	22.0
	College and above	Fathers	19.8
		Mothers	12.2
Occupational status	Farmers or unemployed	Fathers	31.0
		Mothers	40.3
	Semi-professional	Fathers	41.4
		Mothers	28.6
	Professionals	Fathers	25.1
		Mothers	26.4
Monthly family income (Yuan)	Below 3,000	16.1	
	3,000 to 6,000	51.4	
	Above 6,000	32.5	

### 3.2 Common method bias test

Common method bias was assessed using Harman single-factor analysis. The procedure identified nine factors with eigenvalues exceeding 1.0. The primary factor explained 20.23% of total variance, under the 40% critical threshold, indicating no substantial common method bias in this study (Podsakoff et al., 2003).

### 3.3 Descriptive statistics

The descriptive analysis of the study variables was shown in Table 3.

**Table 3:** Descriptive statistics (*N* = 2,588).

**Variables**	**Mean**	**SD**
Neuroticism	2.66	0.67
Maternal care factor	4.17	0.48
Maternal encouraging autonomy factor	3.94	0.72
Maternal control factor	2.07	0.45
Depression	1.80	0.41
SES	-0.01	1.00

### 3.4 Difference test

One-way ANOVA in SPSS revealed that education levels of mothers had significant impact on depression scores: *F*(2, 2585) = 5.305, *p* = 0.005, η^2^ = 0.004. Students whose mothers have an education level below high school have a higher level of depression than students whose mother attained a high school education background (*mean difference* = 1.58, *SD* = 0.49, *p* = 0.001). Additionally, a significant influence of paternal education levels on depression was found: *F*(2, 2585) = 3.628, *p* = 0.027, η^2^ = 0.003. Students whose fathers have an education level below high school have a higher level of depression than students whose fathers attained a education background above college (*mean difference* = 1.30, *SD* = 0.52, *p* = 0.012). No significant impact of maternal occupational status found on depression of students *F*(2, 2463) = 1.345, *p* = 0.261, η^2^ = 0.001. However, paternal occupational status significantly influenced college students' depression levels: *F*(2, 2520) = 3.834, *p* = 0.022, η^2^ = 0.003. Students whose fathers were farmers or unemployed have a higher level of depression than students whose fathers were semi-professionals (*mean difference* = 1.22, *SD* = 0.47, *p* = 0.01), or professionals (*mean difference* = 1.14, *SD* = 0.53, *p* = 0.031). Levene's test indicated a violation of homogeneity of variances *F*(2, 2585) = 5.023, *p* = 0.007; therefore, Welch-corrected ANOVA and Games-Howell *post-hoc* tests were employed. The Welch-corrected analysis revealed significant differences among the three groups *F*(2, 1072.70) = 18.235, *p* < 0.001, effect sizeε^2^ = 0.014. *Post-hoc* comparisons demonstrated that: The below 3,000 RMB/month group had significantly higher depression scores than the 3,000–6,000 RMB/month group (*mean difference* = 1.50, *SD* = 0.60, *p* = 0.034) and the above 6,000 RMB/month group (*mean difference* = 3.46, *SD* = 0.63, *p* < 0.001); The 3,000–6,000 RMB/month group also scored significantly higher in depression than the above 6,000 RMB/month group (*mean difference* = 1.97, *SD* = 0.43, *p* < 0.001).

### 3.5 Correlations

Correlations among variables were presented in Table 4. Depression were found to have a positive correlation with neuroticism (*r* = 0.572, *p* < 0.001); and maternal control factor (*r* = 0.253, *p* < 0.001). Depression exhibited a negative correlation with maternal care factor (*r* = −0.402, *p* < 0.001); and maternal encouraging autonomy factor (*r* = −0.345, *p* < 0.001). Neuroticism showed a positive correlation with the maternal control factor (*r* = 0.253, *p* < 0.001) and negatively associated with maternal care factor (*r* = −0.311, *p* < 0.001) and the maternal encouraging autonomy factor (*r* = −0.272, *p* < 0.001).

**Table 4:** Correlations among variables.

**Variables**	**1**	**2**	**3**	**4**	**5**	**6**
1. Neuroticism						
2. Maternal care factor	-0.32^**^					
3. Maternal encouraging autonomy factor	-0.27^**^	0.54^**^				
4. Maternal control factor	0.22^**^	-0.34^**^	-0.27^**^			
5. Depression	0.59^**^	-0.40^**^	-0.35^**^	0.25^**^		
6. SES	-0.05^**^	0.14^**^	0.07^**^	0.01	-0.08^**^	

** *p* < 0.01; all values were rounded to two decimal places.

### 3.6 Simple moderation analysis

The outcomes of moderation analyses were presented in this section. First, it was found that neuroticism had a significant and positive direct effect on depression (*B* = 0.717, β = 0.571, *p* < 0.001) after controlling for the influence of SES, with the total model accounting for 33.2% of the variance (*R*^2^ = 0.332). Additionally, the influence of SES on depression was found to be significant (*p* < 0.002). Moderation models were used to examine the relationship between college students' depression (dependent variable), neuroticism (independent variable), and three dimensions of maternal parenting practices (moderating variables). The bias-corrected Bootstrapping method was employed to calculate the confidence intervals, with the sampling times were set to 1,000 times, and the confidence interval was set to 95%. The mean, low, and high values of the moderating variables were measured according to MD-1SD, MD, MD+1SD.

#### 3.6.1 Maternal care factor

The results of the hierarchical regression model was presented in the Table 5, which considers neuroticism as an independent variable and college students' depression as a dependent variable. Maternal care factor was entered as moderator. As indicated in Table 5, maternal care was identified as having a negative predictive effect on depression (*p* < 0.001), while neuroticism positively predicted depression (*p* < 0.001). Additionally, the interaction between the independent variable and the moderator was statistically significant, indicating significant moderation (Δ*R*^2^ = 0.001, *p* = 0.046).

**Table 5:** Hierarchical regression model of predictors of depression, considering maternal care (*R*^2^ = 40.63%, *p* < 0.05).

**Variables**	***b***	**95%CI**	**SE**	***t***	***p***	***R* ^2^**
SES	-0.01	[-0.02, 0.01]	0.006	-1.15	0.25	
Constant	1.80	[1.78, 1.81]	0.007	271.88	<0.001	
Neuroticism	0.32	[0.30, 0.34]	0.010	31.35	<0.001	
Maternal care	-0.21	[-0.23, -0.18]	0.015	-13.79	<0.001	
Maternal care × Neuroticism	-0.05	[-0.09, 0.00]	0.022	-2.11	<0.05	40.63%

**Figure 2:**
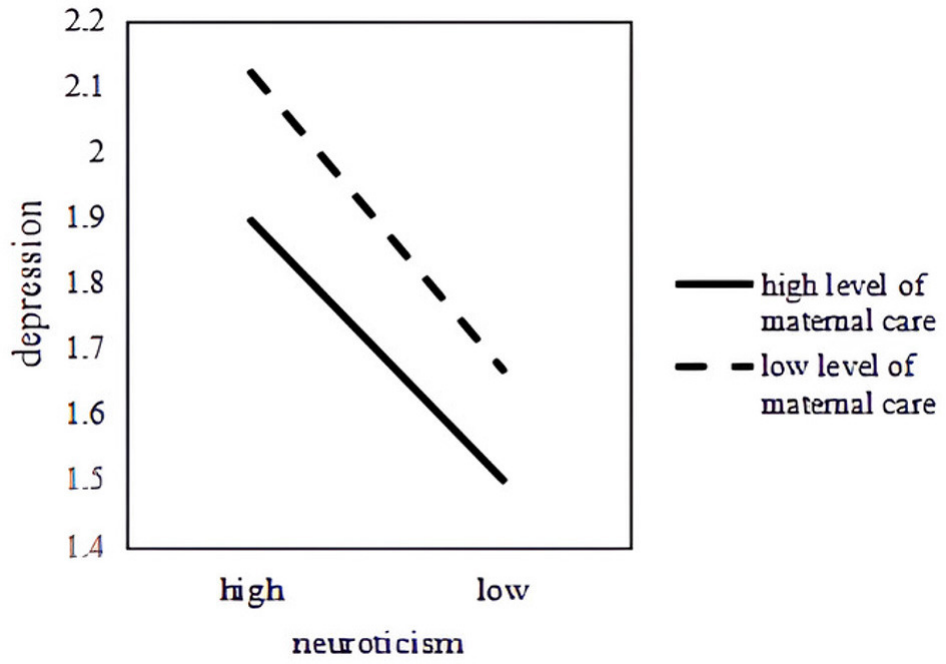
The moderation effect of maternal care.

Subsequently, the association between neuroticism and college students' depression was further investigated using simple regression slopes under different levels of maternal care (Figure 2). As maternal care increases, the relationship between depression and neuroticism weakened.

#### 3.6.2 Maternal encouraging autonomy factor

The results of the hierarchical regression model were presented in Table 6. Neuroticism was considered as an independent variable, with depression serving as a dependent variable. Maternal encouraging autonomy was included as a moderator. As shown in Table 6, maternal encouraging autonomy negatively predicted depression (*p* < 0.001), while neuroticism positively predicted depression (*p* < 0.001). The interaction between the independent variable and the moderator was statistically significant for the model, indicating that the moderation effect was significant (Δ*R*^2^ = 0.0046, *p* = 0.004).

**Table 6:** Hierarchical regression model of predictors of depression, considering maternal encouraging autonomy (*R*^2^ = 39.16%, *p* < 0.05).

**Variables**	***b***	**95%CI**	**SE**	***t***	***p***	***R* ^2^**
Constant	1.79	[1.78, 1.81]	0.007	273.25	<0.001	
Neuroticism	0.33	[0.31, 0.35]	0.010	33.24	<0.001	
Maternal encouraging autonomy	-0.11	[-0.13, -0.09]	0.009	-11.75	<0.001	
Autonomy × neuroticism	-0.06	[-0.08, -0.03]	0.013	-4.39	<0.001	39.16%

**Figure 3:**
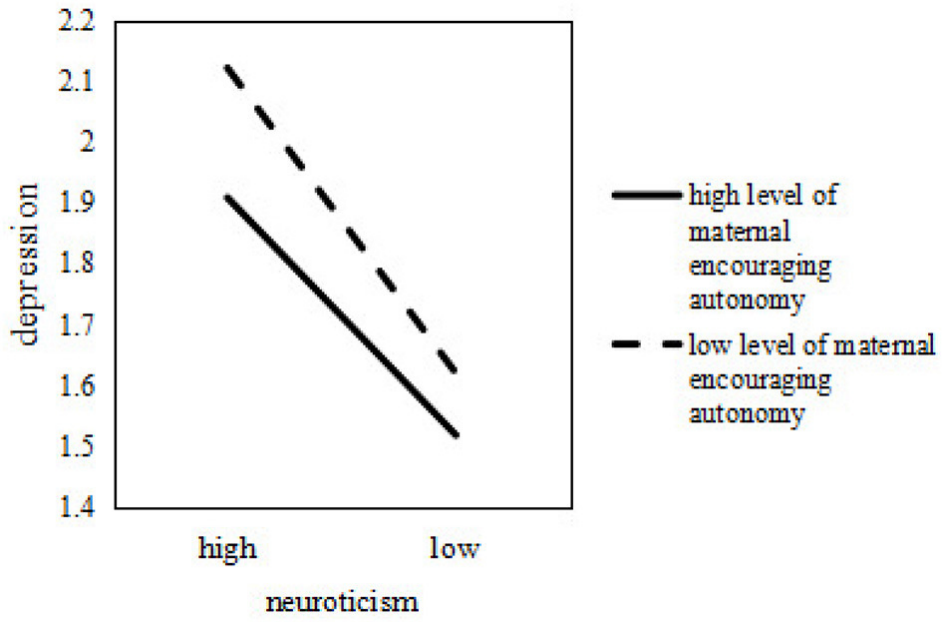
The moderation effect of maternal encouraging autonomy.

It was subsequently examined how the relationship between depression and neuroticism among college students varied at different levels of maternal encouraging autonomy. It was shown in Figure 3 that as maternal encouraging autonomy increased, the relationship between neuroticism and depression diminished.

#### 3.6.3 Maternal control factor

The results of the hierarchical regression model that considered maternal control factor as moderator and neuroticism as an independent variable was presented in the Table 7. Depression was entered as the dependent variable. As shown in Table 7, maternal control positively predicted depression (*p* < 0.001) and neuroticism was found to have a positive predictive effect on depression. (*p* < 0.001). The interaction between independent variable and the moderator was statistically significant for the model, indicating significant moderation (Δ*R*^2^ = 0.0019, *p* = 0.038).

**Table 7:** Hierarchical regression model of predictors of depression, considering maternal control (*R*^2^ = 36.53%, *p* < 0.05).

**Variables**	***b***	**95%CI**	**SE**	***t***	***p***	***R* ^2^**
Constant	1.80	[1.78, 1.81]	0.007	270.95	<0.001	
Neuroticism	0.35	[0.33, 0.37]	0.010	34.59	<0.001	
Maternal control	0.10	[0.07, 0.13]	0.015	6.67	<0.001	
Maternal control × neuroticism	0.06	[0.02, 0.11]	0.023	2.76	<0.01	36.53%

**Figure 4:**
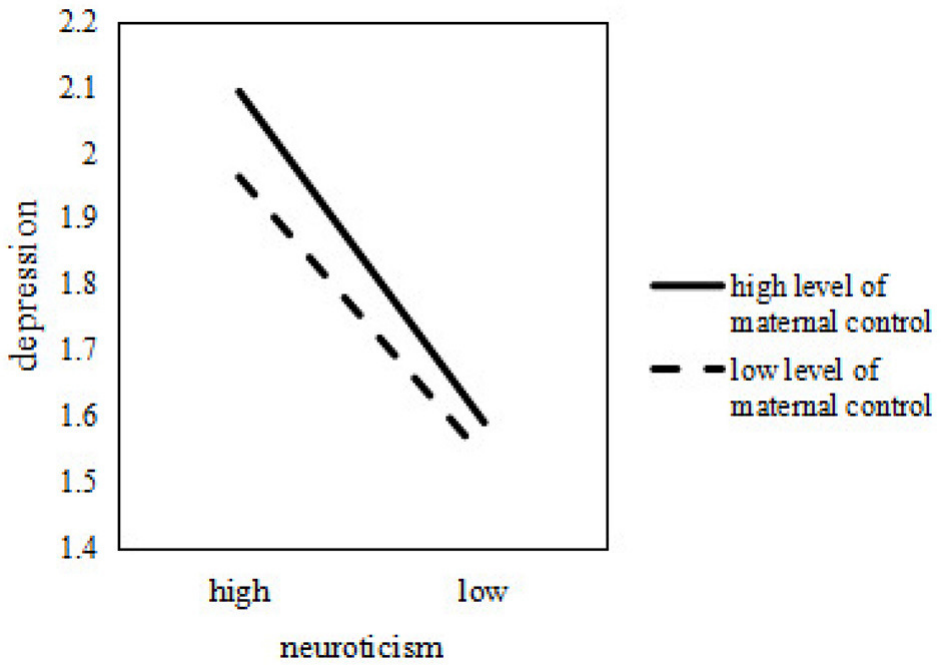
The moderation effect of maternal control.

It was subsequently examined how the association between depression and neuroticism among college students differed across various levels of maternal control. The findings from the simple regression slope analysis were presented in Figure 4. As depicted, with increasing maternal control, the association between neuroticism and depression strengthened.

## 4 Discussion

Based on available research and Diathesis-Stress Model, a moderation model was constructed in this study to examine whether neuroticism significantly predicts depression in a substantial Chinese college sample and how neuroticism interacts with maternal parenting practices to influence depression. The results indicate that increased maternal care or maternal encouraging autonomy lessens the negative impact of neuroticism on depression, while greater maternal control enhances the predictive power of neuroticism for depression. This investigation contributes theoretically by elucidating personality-family environment interactions in mental health outcomes, advancing cross-cultural applications of the diathesis-stress model in Chinese college students.

The present study aligns with prior research in identifying neuroticism as a robust predictor of depression (Kotov et al., 2010; Wilks et al., 2020; Yang and Koo, 2022; Yang et al., 2023). This trait correlates with increased susceptibility to negative affective states—including sadness, anxiety, and anger (Yang and Koo, 2022)—and elevated perceived stress levels (Cui, 2023). Individuals exhibiting high neuroticism typically demonstrate impaired emotional regulation capacities and heightened sensitivity to environmental stressors (Lahey, 2009). Chronic emotional dysregulation, a recognized depression precursor (Joormann and Stanton, 2016), manifests neuro-biologically through stress-induced reduction in prefrontal cortex activity, a putative mechanism underlying depressive pathology (Cui et al., 2024).

Parenting practices are believed to be vital in shaping the development and progression of depression (McLeod et al., 2007; Kaslow et al., 1994). Consistent with prior findings, maternal control demonstrated a significant positive association with depression levels (Chubar et al., 2020). Parental misunderstanding may contribute to heightened feelings of helplessness, loneliness, and insecurity, thereby amplifying vulnerability to depressive states (Kaslow et al., 1994). Empirical evidence and meta-analysis consistently indicate that adverse parenting practices elevate depression risk by reinforcing maladaptive cognitive styles and undermining self-worth (Enns et al., 2000; Diaconu-Gherasim et al., 2017). In contrast, maternal encouraging autonomy and maternal care revealed negative correlations with depressive symptoms in the current investigation. Adolescents raised by emotionally supportive and accepting parents typically exhibit enhanced emotional stability, self-acceptance, and life satisfaction (Kaslow et al., 1994). Research has further established that constructive parenting practices may serve as protective mechanisms against the emergence of adolescent rumination and subsequent depressive symptomatology (Gaté et al., 2013).

This study highlights the dynamic, context-dependent nature of personality's role in depression. Aligning with family systems theory, which posits that individual development arises from bidirectional person-environment interactions (Beardslee et al., 2003; Bronfenbrenner and Ceci, 1994; Cox and Paley, 1997), we identify maternal parenting practices—as core family system components—significantly modulate the psychological functioning of the offspring (Kaslow et al., 1994). Neurotic individuals, characterized by heightened environmental sensitivity (Lahey, 2009), exhibit amplified neuroplastic responsiveness to the emotion-regulatory effects of maternal parenting. The current study provides initial empirical support for the moderating role of maternal care, maternal encouraging autonomy, and psychological control in shaping neuroticism-depression associations within this cohort.

The detrimental impact of neuroticism on depression risk is markedly potentiated among individuals enduring chronic environmental adversities (Kendler et al., 2004). Aligning with the diathesis-stress model's premise—that psychopathology emerges from dynamic interactions between intrinsic vulnerabilities and contextual stressors (Monroe and Simons, 1991)—our observed moderation effects substantiate this framework. Specifically, neuroticism (a trait-based diathesis heightening stress reactivity) synergistically interacts with persistent environmental threats, exemplified here by suboptimal maternal parenting practices, to exacerbate depression. The possible neurobiological explanation is that individuals with elevated neuroticism (a key diathesis) demonstrate exaggerated amygdala responsiveness to aversive stimuli and diminished prefrontal regulatory capacity, resulting in compromised emotion modulation (Disner et al., 2011). Exposure to adverse environmental conditions (e.g., parental psychological control) exacerbates these neural vulnerabilities, thereby elevating susceptibility to depressive symptoms. Conversely, nurturing contexts (e.g., maternal care and maternal encouraging autonomy) may mitigate innate diathesis by fostering cognitive reappraisal skills and enhancing adaptive coping strategies (Southwick and Charney, 2012). Crucially, this study extends beyond traditional deficit-focused paradigms by empirically validating protective mechanisms of maternal care and the maternal encouraging autonomy, which shed light on the future prevention and intervention of depression in youth.

Neurotic individuals exposed to suboptimal parenting practices—particularly psychological control—face elevated risks of developing depression. Psychological control operationalizes through relational manipulation (e.g., guilt induction, love withdrawal), emotionally charged criticism (e.g., shame/disappointment expression), and intrusive monitoring (e.g., over-protectiveness, possessiveness) (Tugnoli et al., 2022). This controlling parenting style induces chronic stress, triggering hypothalamic-pituitary-adrenal (HPA) axis hyperactivity—a well-established neurobiological marker of depression (Cui et al., 2024). Mechanistically, maternal control fosters maladaptive outcomes including learned helplessness, chronic insecurity, and social isolation (Tugnoli et al., 2022), while simultaneously impairing self-efficacy development (Wu et al., 2023) and social competence acquisition (Yu et al., 2023; Ren, 2023). This may result in the frustration of basic psychological needs, which, synergistically interacting with the inherent difficulty in fulfilling psychological needs among university students with high neuroticism (Xiao, 2022), constitutes a compounding risk factor for mental health. These deficits culminate in social adaptation impairments (Zhao, 2022) and interpersonal relationship disruptions (Zheng et al., 2023), collectively exacerbating depression vulnerability. Notably, neurotic individuals' inherent emotional dysregulation and environmental hypersensitivity (Cui et al., 2024) potentiate these pathogenic effects through stress amplification mechanisms.

This study elucidates that neurotic college students may avoid depressive outcomes when raised within supportive, nurturing, and autonomy-promoting familial environments. Self-determination theory identifies three fundamental psychological needs—autonomy, competence, and relatedness—which are essential for psychological wellbeing (Deci and Ryan, 2000). Frustration of these needs is regarded as a significant risk factor for depression (Liu et al., 2024). Neurotic individuals' characteristic deficits in fulfilling these needs (Xiao, 2022) heighten depression vulnerability, a pathway modifiable through maternal parenting practices that actively fulfill or frustrate these psychological requirements. Empirical evidence confirms parental autonomy support enhances basic need satisfaction, whereas psychological control exacerbates need frustration (Wei et al., 2022). High-quality maternal parenting fosters self-acceptance, emotional stability, and life contentment (Wu et al., 2023), thereby mitigating the pathogenic effects of neuroticism. Familial social support may prevent depression by enhancing self-esteem to reduce dysfunctional attitudes and promoting autonomy-driven social network expansion, alleviating neuroticism-related anxiety through peer support systems (Cui et al., 2024; Zhao, 2025; Wei et al., 2022).

The current findings indicate that maternal control amplifies the predictive effects of neuroticism on depression, though this moderation demonstrates modest effect magnitudes. Notably, maternal encouraging autonomy—compared to maternal care and maternal control—emerges as the most potent moderating factor. This phenomenon arises from two possible mechanisms. Firstly, physical distance between college students and their families makes it more difficult for maternal control to affect them psychologically. Secondly, college students allocate most of their time to school rather than their parents, the importance of peer relationships is rising, causing the influence of parenting practices to diminish.

The pronounced moderating effect of maternal encouraging autonomy observed in this study may be explained through multiple empirically supported pathways. First, the transition to independent adulthood requires college students to navigate complex daily and consequential decisions—a process particularly challenging for neurotic individuals exhibiting impaired decision-making capacities under stress (Sălceanu et al., 2024). Autonomy-supportive parenting mitigates this vulnerability by enhancing competence to make decisions (Sălceanu et al., 2024), thereby reducing stress accumulation from maladaptive choices and subsequent depression risk (Monroe and Simons, 1991). Second, neuroticism-related emotional dysregulation (Lahey, 2009) is counterbalanced through the capacity of maternal encouraging autonomy to foster self-exploration, emotional acceptance, and self-affirmation. Empirical evidence from the study in Chinese college students demonstrates that parental autonomy support inversely correlates with depression through self-esteem enhancement (Tan et al., 2024). Specifically, maternal encouraging autonomy enables individuals to cultivate non-judgmental awareness of internal states, develop adaptive emotional schemas, and achieve self-approval through competence validation (Tan et al., 2024), which could be one of the possible explanations of the protective effects of maternal encouraging autonomy.

The differential analysis between the three dimensions of SES and depression revealed interesting findings. First, both paternal and maternal educational levels were negatively correlated with depression, which aligns with previous studies (Zhao et al., 2015; Zhao, 2017). This phenomenon may be attributed to the association between lower parental education and adverse parenting styles (Zhang, 2016). Only the paternal occupational status showed a significant impact on the depressive symptoms of college students, which may stem from the fact that fathers serve as the primary economic providers for households (Zhang et al., 2024). Consequently, paternal occupation is more strongly tied to total monthly household income, which has been shown to impact depression significantly. Students from families earning less than RMB 3,000 per month showing significantly higher depression levels than other income groups. Possible explanations are that low family income heightens students' financial stress (Yan-hong et al., 2014), and that financially limited mothers' capacity to provide autonomy support and emotional warmth (Vreeland et al., 2019), potentially exacerbating depression risk in neurotic students.

### 4.1 Implication

The present study has several strengths. The findings further concluded that both individual factors and family factors provide initial evidence of the influencing mechanism of depression. Methodologically grounded in the diathesis-stress framework, this research advances theoretical precision for targeted prevention, such as early psychological and family counseling for neurotic individuals exposed to adverse parenting, to disrupt depression pathways. These contributions enhance both the scientific rigor and systemic application of the diathesis-stress model in depression research. Notably, it was observed that maternal encouraging autonomy could effectively weaken the prediction effect of neuroticism on depression compared with maternal care and maternal control, which implies important implications to intervention in universities. While familial parenting styles are challenging to modify, our results suggest promoting autonomy among college students. To prevent depression in college students, we can design educational materials on personality theories and positive parenting practices for broader dissemination.

Additionally, while previous studies tended to examine adolescents, few have explored college students. College students represent a developmentally vulnerable population characterized by elevated depression prevalence and suicide risk (Gao et al., 2020). By utilizing a large-scale Chinese college sample (*N* = 2, 588), this study enhances ecological validity while establishing robust generalizability of the findings with immediate preventive implications. The significant socioeconomic gradient in depression outcomes necessitates institutional prioritization of financial aid programs and mental health subsidies for economically disadvantaged students. Furthermore, during psychological counseling or mental health interventions, clinicians should evaluate two key factors: students' personality traits (e.g., neurotic tendencies) and historical parenting experiences. Students exhibiting elevated neuroticism and exposure to highly controlling maternal parenting practices warrant prioritized intervention due to compounded risk profiles, which target on enhancing emotional regulation capacities through mindfulness-based stress reduction techniques.

### 4.2 Limitation

Despite these strengths, several limitations merit our attention. First, all participants were recruited solely from China, potentially restricting the applicability of the conclusions; thus, cross-cultural studies are needed. Second, the current study relied on single-informants for assessing depression, neuroticism and maternal parenting practices. Utilizing multi-method assessments in future studies can yield a more comprehensive understanding. In addition, 80% of the participants were female. Mother-child trust and communication levels are significantly higher among Chinese female college students than among male students (Yin et al., 2021). Therefore, the precision of the findings might be affected. Furthermore, it is essential to further investigate this interaction by considering paternal parenting practices. Finally, the risk of depression might exist in the adolescent and earlier stages of individuals, only exhibit themselves during the university period. Therefore, considering the uniqueness of the parenting practice factor, a longitudinal study tracking from the adolescent period is needed in the future.

## 5 Conclusion

The current study examined the interaction between neuroticism and maternal parenting practices, shedding light on their effects on depression among Chinese college students. Maternal parenting practices were found to act as moderators in this relationship. The results showed that when exposed to high maternal care or high maternal encouraging autonomy, the predictive effect of neuroticism on depression was reduced. However, the predictive effect was strengthened when subjected to high maternal control. These outcomes proved the vital effects of maternal parenting practices. Our findings also provided initial pieces of evidence regarding the influencing mechanisms of depression by both personality and maternal parenting practices. This finding also extended the diathesis-stress model and offers critical implications for interventions and preventions.

## Data Availability

The raw data supporting the conclusions of this article will be made available by the authors, without undue reservation.
